# Nurses’ Readiness to Undertake Controlled Negative Pressure Therapy in the Treatment of Chronic Wounds—Research Report

**DOI:** 10.3390/ijerph20043388

**Published:** 2023-02-15

**Authors:** Joanna Przybek-Mita, Dariusz Bazaliński, Maria Teresa Szewczyk, Daria Kardyś, Bartosz Mańkowski, Paweł Więch

**Affiliations:** 1 Institute of Health Sciences, College of Medical Sciences, University of Rzeszów, 35-959 Rzeszów, Poland; 2 Postgraduate Nursing and Midwifery Education Centre, 35-083 Rzeszów, Poland; 3 Podkarpackie Specialist Oncology Centre, Specialist Hospital in Brzozów Father B. Markiewicz, 36-200 Brzozów, Poland; 4 Department of Perioperative Nursing, Department of Surgical Nursing and Chronic Wound Care, Collegium Medicum in Bydgoszcz, Nicolaus Copernicus University in Torun, 85-821 Bydgoszcz, Poland; 5 Department of Hematology, Clinical Provincial F. Chopin Memorial Hospital No. 1, 2 Szopen Street, 35-055 Rzeszów, Poland; 6 General Surgery and Multi-Organ Disorders Ward, Multidisciplinary Municipal J. Struś Memorial Hospital in Poznań, 61-285 Poznań, Poland; 7 Craniofacial Surgery Clinic of Medical University in Poznań, 61-701 Poznań, Poland; 8 Departmenet of Nursing, Institute of Health Protection, State University of Applied Sciences in Przemyśl, 37-700 Przemyśl, Poland

**Keywords:** nurse, perception, NPWT, chronic wound

## Abstract

Local wound treatment with negative pressure wound therapy (NPWT) shortens the healing process but requires the supervision of trained medical personnel for administering the therapeutic procedures. Professional supervision and control of the effectiveness of NPWT, as well as education conducted by nurses, are of particular importance for therapeutic and caring processes, both in hospital and at home. The aim of the study was the assessment of the perception of NPWT by certified nurses in the topical treatment of chronic wounds. The study was conducted using the method of estimation and a diagnostic survey with the proprietary NPWT perception questionnaire: 495 subjects were enrolled in the study and 401 respondents aged 25–67 years qualified for statistical analysis. Despite their experience and competence, the respondents critically assessed their knowledge, pointing to an average subjective level of knowledge related to wound treatment and a low level of knowledge related to NPWT. Most of the respondents had no experience of independent treatment using this method. The data obtained from the questionnaire clearly indicate theoretical preparation and high motivation to undertake activities related to the implementation of NPWT in their own practice. Low readiness values suggested that the subjects did not have the resources or the ability to implement the method. The perception of NPWT in the surveyed group of nurses was determined by numerous factors, including self-assessment of their own knowledge, motivation and readiness to use NPWT. A high level of NPWT perception was noted, despite low motivation related to the availability and knowledge of the method. Theoretical knowledge is insufficient to implement innovative methods in local wound treatment. Practical skills and motivation are indispensable elements that should be met during the training and education of nurses in the field of wound care.

## 1. Introduction

Wound healing is frequently a long-term process burdened with the risk of infection and failure of planned therapeutic measures [1]. The debridement of devitalized tissues, infection control, the evacuation of exudate and the stimulation of wound regeneration are the basic assumptions of the TIME(RS) strategy [2] based on the concept of wound bed preparation (WBP) proposed and implemented by Sibbald et al. [3,4]. Although numerous methods available in local wound therapy in inpatient and outpatient care encompass primarily medical devices with antiseptic properties that promote tissue reorganisation processes [5,6], negative pressure wound therapy (NPWT) still deserves attention [7]. NPWT is a minimally invasive, widely recognized physical method that supports the local treatment of open wounds of various etiologies. In the treatment of chronic wounds, the application of NPWT offers a wide range of therapeutic possibilities, especially for home settings in the case of deep wounds with signs of infection and high exudate. The positive effect on wound healing is determined by four basic mechanisms (macrodeformation, microdeformation, exudate management and changes in the wound environment) as well as secondary mechanisms (neurogenesis, angiogenesis, inflammation modulation and changes in biological load) [8,9]. Local treatment with NPWT allows the wound healing process to be shortened but requires the supervision of trained medical personnel for administering the therapeutic procedures [10,11,12,13]. Maintaining continuity of care with a recently introduced method in our facilities method such as NPWT reduces the risk of complications and shortens the wound healing time. Legal possibilities allow the implementation and practice of modern therapies by nurses. Professional supervision and follow-up of the effectiveness of NPWT, as well as education, are of particular importance in therapeutic and caring processes, both in hospital and also for home-based therapy where the caring function can be performed by the family (caregivers) of the patient. Telephone follow-up and teleconsultation with the person in charge (nurse or doctor) using audiovisual devices should be considered whenever there is no possibility of direct inspection for potential therapeutic problems [14]. So far, no in-depth studies on the assessment of the perception of NPWT therapy in nurses have been conducted worldwide. Considering the effectiveness of the method in the local treatment of chronic wounds, our study aimed to establish the perception of nurses’ readiness to use NPWT for the treatment of chronic wounds in outpatient care. The use of NPWT in local wound treatment procedures is an increasingly frequent therapeutic option recommended by scientific societies. In order to maintain the continuity of treatment and minimize the potential risk of infection, it is preferable to treat the wound in a hospital setting and to continue treatment in outpatient care [15]. Standardized therapeutic protocols and specialist care for patients with chronic wounds, conducted by trained medical personnel, positively determine the quality of life of patients by reducing the risk of complications, improving their ability for self-care and, in principle, shortening the duration of therapy without the need for frequent hospitalization. In Poland, legislation permits nurses to make independent decisions in the process of treating wounds. The literature on wound treatment draws attention to the preference mainly for medical devices (active dressings and hydrogels, including antiseptic devices) in hospital care, while the method of NPWT is still in the implementation and recommendation phase [16,17]. Ensuring continuity of care for a patient with a wound using innovative treatment methods (NPWT; maggot debridement therapy, MDT; and hyperbaric oxygen therapy, HBOT) would probably improve treatment outcomes. The results of the studies clearly indicate the usefulness of NPWT in the wound healing process [7,9] and, thus, for the method to be implemented in clinical practice it should be made known and available to professionals dealing with wound therapy. Reports and meta-analyses from recent years clearly indicate the advantages of NPWT and the increasing possibilities for its use in reducing the costs and risk of wound infection [18,19].

## 2. Materials and Methods

### 2.1. Ethical Considerations

The study protocol was approved by the Bioethics Commission at the University of Rzeszow (Resolution no. 2018/01/07f, 7 January 2018). Moreover, the guidelines of the Helsinki Declaration were observed in the course of the conducted study. Participants were informed of the purpose of the study and the fact that they could withdraw at any point without giving a reason.

### 2.2. Study Design

In 2021, the consensus on care and education during NPWT [20] at home became the inspiration to create the methodological assumptions of this study. In the present study, which is likely to be a pioneering scientific endeavor, the authors focused on the perception (knowledge, motivation and readiness) of NPWT in a group of qualified nurses dealing with the prevention and treatment of chronic wounds.

#### 2.2.1. Phase 1: Exploring the Theoretical Evidence

A review of the literature in the PubMed database from 2015 to 2022 on the issues of wound treatment with NPWT was carried out using the following keywords: NPWT and chronic wound. Out of 423 selected works, 39 studies were selected, on the basis of which the NPWT factors in the local treatment of chronic wounds were preliminary developed. Based on the assumptions of the study regarding knowledge, motivation and readiness, a database of 40 factors related to the assumptions and issues of NPWT was prepared.

#### 2.2.2. Phase 2: Developing a Research Tool

In the second stage of the study, a questionnaire was developed, based on the prepared issues related to NPWT, with 10 nurses and two surgeons asked to provide their opinions on the prevention and treatment of wounds with NPWT. The reviewers were experienced wound care clinicians. Doctors and 4/10 nurses had documented scientific activity in this field. A total of 40 factors (20 on NPWT knowledge, 10 on motivation to implementation NPWT and 10 on readiness to implement NPWT) were formulated based on the issues raised in scientific publications. The most accurate ones were selected, which were most often indicated as crucial by the respondents. Based on the opinions of the respondents, who mostly stated that “*the implementation of NPWT in the group of nurses may be successful if the mentoring system is implemented and the nurse can get support with the implementation of the method*”. The second part was the NPWT perception questionnaire developed for the purpose of the study, consisting of 20 items distributed in three subscales (domains): domain I (knowledge), 10 items; domain II (motivation), 5 items; and domain III (readiness), 5 items. Answers to individual questions were developed on the Likert scale (strongly disagree; disagree; have no opinion; agree; and strongly agree) with scoring from 1 to 5. The prepared research tool (NPWT perception questionnaire) was tested in a pilot study on a group of 20 randomly selected nurses; no issues in comprehension of the items were found and the form of the study was not questioned (Table 1).

Respondents could obtain a minimum of 10 points and a maximum of 100 points. The reliability of the NPWT perception questionnaire was assessed in individual domains and in total at the Cronbach alpha level of 0.92. The key for evaluation of individual scales was developed in the course of the statistical analysis (Table 2) and the sum of the individual subscales gave the overall result of NPWT perception in the studied sample.

#### 2.2.3. Phase 3: Proper Study in a Group of Certified Nurses

The actual research was carried out during the period from February to April 2022 on a group of 495 nurses (representing 75.5% of all participants) participating in training courses in the field of wound treatment conducted by Urgo Medical Polska in the Polish cities of Katowice, Rzeszów, Radom, Bydgoszcz and Kielce. Each of the respondents voluntarily consented to the survey. After presentation of the subject of the study, the respondents were handed out previously prepared questionnaires and the purpose, principles of the study and the specified time were explained. The questionnaires were completed before training in no more than 15 min. Then, each completed sheet was thoroughly verified on the basis of the inclusion and exclusion criteria, which allowed for the collection of reliable data. Ultimately, 401 questionnaires qualified for statistical analysis. A total of 91 questionnaires were rejected: 16 with missing or incomplete data on functioning in the profession or incomplete answers on the NPWT perception questionnaire; and 75 due to participants’ resigning from the study.

The incompleteness of the data in the questionnaire was the lack or incompleteness of sociodemographic data related to functioning in the profession, incomplete answers in the NPWT perception questionnaire. Due to the fact that the study was voluntary, some of the qualified participants asked to withdraw from the study, noting the level of their incomplete knowledge of the method and the inability to implement it in practice.

### 2.3. Participants

The study involved nurses with qualifications and competence in the prevention and treatment of wounds and conducting professional activity with patients in out-of-hospital care.

### 2.4. Research Tools

The study was carried out by means of the estimation method and a diagnostic survey using the proprietary questionnaire, which consisted of two parts. The first part contained a sociodemographic record additionally enriched with two statements referring to the level of independence, experience and knowledge (scale of 0–10: 0, lack of knowledge; 10, high level of knowledge); the statements concerned a self-assessment of their general knowledge of wound care and knowledge related to NPWT.

The second part of the research tool was the NPWT perception questionnaire, which included 20 items.

### 2.5. Enrolment to the Study

**Figure 1:**
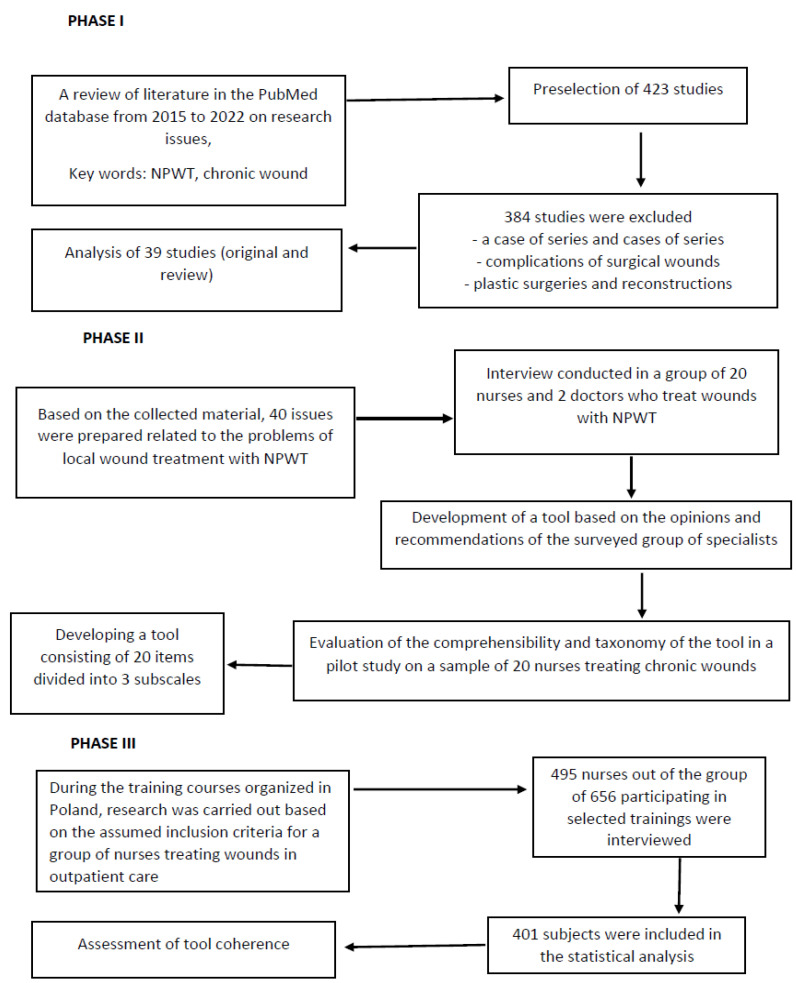
Flow chart concerning the organization and conduct of the study.

The inclusion criteria were voluntary consent, having the right to practice as a nurse in Poland, being authorized to treat wounds (at least a specialist course) and activity in a given field related to the treatment of wounds for at least 6 months. People who did not meet certain conditions were excluded from the study group. The main criteria for exclusion from the study were the lack of consent to complete the questionnaire and a lack of interest in the NPWT method despite having qualifications for the prevention and treatment of wounds. The process of tool construction and research organization is illustrated in Figure 1.

### 2.6. Statistical Analysis

Statistical analysis of the collected data was performed using Statistica 13.3 software (StatSoft). The analysis was performed using Spearman’s rank correlation, which is a nonparametric test. Its selection was conditioned by failure of the data to meet the basic assumption of parametric tests (i.e., compliance of the distribution of the studied variables with a normal distribution or homogeneity of variance). The consistency of the distributions with a normal distribution was verified with the Shapiro–Wilk test, whereas the homogeneity of variance was assessed with Levene’s test. These results were supplemented with the results of the significance test of the correlation coefficient (p), which made it possible to assess whether the relationship found in the sample reflects a more general relationship prevailing in the entire population or is just a matter of chance. The level of statistical significance was adopted as *p* < 0.05.

## 3. Results

### 3.1. Characteristics of the Study Group

A total of 401 fully completed research questionnaires were included for statistical analysis. The study enrolled 391 women (97.5%) and 10 (2.5%) men aged 25–67 years who qualified for statistical analysis. The mean age of the respondents was 44.96 years. The collected data are presented in Table 3.

### 3.2. Experience in NPWT

Each of the respondents had experience in the field of prevention and treatment of wounds (100.0%). The subjects were asked to assess their general level of knowledge about wound care and NPWT on linear scales from 0 to 10 points (0, lack of knowledge; 10, high level of knowledge). The respondents obtained an average score of 5.77 points (SD = 2.26). Only a small number of people scored 0 points, whereas most scored 10 points. Subjective knowledge of wound treatment and NPWT was similarly assessed on a 10-point scale, where the average score of the respondents was 3.95 points (SD = 2.71). These results are related to the fact that as many as 53.6% of respondents had no experience in applying a vacuum dressing and only 12.0% applied the NPWT dressing on their own as part of the therapeutic and care activities (Table 4).

### 3.3. Nurses’ Perception of NPWT Therapy in the Treatment of Chronic Wounds

NPWT perception in the study group was assessed on the basis of 20 items grouped in three domains (knowledge, motivation and readiness). It was assumed that perception in the study group would be at an average level. The total score range in the NPWT tool was 20–100 points, with an average score of 75.77 (SD = 12.72). The lowest score was 37 and the highest was 100. These data indicate a high level of NPWT perception in the studied sample (Table 5).

Most respondents scored highly (59.1%) in the domain that assesses knowledge. For the motivation domain, most scores were average (47.6%) or high (38.2%). As far as readiness to apply the therapy, many (28.2%) respondents were not likely to implement NPWT or manifested a low readiness to use it (26.9%). Only 18.5% of the nurses declared a high readiness to implement NPWT. The data clearly indicated nurses’ theoretical basis and high motivation to take actions related to use of the method in practice. However, low values for readiness suggest that the respondents did not have the resources or the possibility of implementing the method in their professional practice. Detailed data are presented in Table 6.

### 3.4. Perception of NPWT and Selected Variables

In the course of statistical analysis, data on professional experience, education qualifying for wound treatment, mentoring support, place of practice and the perception and readiness to undertake NPWT were compiled. Statistical analysis showed a strong correlation (*p* < 0.001) between support related to NPWT implementation and concerns about undertaking this method. It was observed that the more the respondents had support, the lower their fears that NPWT could harm a patient with comorbidities (e.g., diabetes or heart failure), that they could wrongly decide to use NPWT and a health risk would occur, and that the patient could destroy expensive equipment and they would have to bear the costs. It was assumed that people with greater knowledge about NPWT were also more motivated and more ready to implement NPWT (domain I vs. II and III). The study confirmed the existence of a relationship between the level of knowledge and the level of motivation and readiness to implement NPWT (*p* < 0.001). These relationships were relatively strong and had a positive direction, which indicates that people with greater knowledge on NPWT showed greater motivation and readiness to implement this method in practice (Table 7).

In the course of statistical analysis, the correlation between age and professional education in wound treatment and the level of NPWT perception was checked. It was assumed that those with greater work seniority and those with a higher level of nursing education would present a higher level of NPWT perception; however, this relationship was not confirmed here (*p* > 0.05) (Table 8). On the other hand, the presence of a statistically significant difference was confirmed between readiness (domain III) to treat wounds and completing a specialist course on wound treatment (*p* = 0.009). Nurses who had completed a wound treatment course (Table 9) showed greater readiness.

It was also shown that people working in surgical wards had a higher level of readiness to implement NPWT compared to those not working in such wards (*p* < 0.001) (Table 10).

## 4. Discussion

The use of advanced therapeutic procedures in the treatment of chronic wounds, reducing the risk of complications/infection and accelerating the repair processes, requires trained medical personnel who, in addition to having knowledge and clinical experience, provide education [21]. Preparation in the course of postgraduate education is one of the conditions for gaining the knowledge, skills and competence to implement NPWT. The study undertaken was aimed at assessing the perception of nurses dealing with the prevention and treatment of wounds towards the implementation of NPWT in out-of-hospital care. NPWT can be carried out as an extension of hospital treatment in the home environment and preparing the patient for local treatment with this method can be performed effectively at home by nurses. A meta-analysis by Xie et al. verified the effect of NPWT on wounds compared to conventional dressings after surgical wound closure. Wound therapy with NPWT was characterized by a significantly lower incidence of deep infection of the operated site and wound dehiscence compared to conventional methods using active dressings. The authors did not note a significant effect on the length of hospital stay compared to conventional methods [19].

Huang et al. indicate the developed consensus on the comprehensiveness of healthcare and education during NPWT at home for patients with chronic wounds [20]. Treatment using controlled negative pressure involves connecting the patient to the equipment for 24 h a day and checking the tightness, which results in certain limitations in everyday life. This obliges the patient and their family to actively participate in the treatment process, but it creates better conditions for wound healing and minimizes treatment costs in the long term [7,22,23].

The benefits of using NPWT in the treatment of chronic wounds favor the implementation and recommendation of this method in order to accelerate the healing process. However, obtaining the desired therapeutic effects closely correlates with the knowledge, skills and readiness to use NPWT in everyday wound treatment practice. Until now, there have been no publications assessing the theoretical and practical preparation and readiness to implement NPWT by Polish nurses, which proves the innovative concept of this study. Bazaliński et al. conducted studies on the readiness to implement biodebridement with the use of *Lucilia sericata* larvae, which indicated that the lack of knowledge, skills and experience in the treatment of wounds using MDT could result in a reluctance to implement this form of therapy. It was shown that self-assessment of the general principles of wound treatment did not increase the self-assessment of knowledge in the field of biological therapy, while the high values obtained for self-assessment on the use of MDT in wound treatment increased the level of readiness to use biotherapy, which in turn positively influenced the perception of MDT [24]. Other experts showed that the initial reluctance was overcome by favorable treatment outcomes [25,26,27].

Analysis of the collected material shows some similarities regarding the readiness to implement MDT and NPWT. Similarly, the study demonstrated a low level of experience of nurses in the treatment of wounds with the use of NPWT: only 12.0% of respondents independently conducted wound prevention and treatment with the use of NPWT, and 20.9% assisted in such procedures, which were performed by doctors. Nevertheless, the conducted survey showed great interest and readiness despite low motivation to introduce the method. Moreover, a positive correlation was confirmed between support in the conduct and implementation of NPWT for the treatment of chronic wounds and emerging concerns regarding the use of this method. Those subjects who have been mentored in the field of NPWT showed much less concern over possible complications, making the wrong decision to implement the therapy or incurring costs related to failure. A clinical practice based on mentoring, which encourages professional independence and decision-making, creates wide opportunities for the development of nursing, providing high-quality care and influencing therapeutic professionalism [28]. Concerns related to the wrong decision to implement NPWT, resulting in the possibility of endangering the patient’s health and thus legal liability, have grounds in the group of respondents. They suggest high concern for potential patients, where the implementation of specialized local treatment methods, in the absence of experience, could be dangerous and create a risk of complications. These concerns may also result from insufficient knowledge related to NPWT, decision-making dilemmas and professional and legal responsibility in the activities that a nurse may independently undertake in the treatment of wounds. A modern, effective and advanced but still not very common therapeutic method could be more readily led by numerous nurses in Poland if they had the support of experienced clinicians, who would provide them with a sense of security and guidance in the initial steps taken on the path of innovative wound treatment. Standardization and the development of guidelines for the care of patients with a wound treated using NPWT would be the next milestone in advanced practice out of hospital. These observations should be analyzed in more detail in a well-thought-out and future-oriented study.

It is also worth noting that a significant percentage of respondents indicated long-term home care (14.5%) and home hospice (11.5%) as the place to perform professional activities: nurses characterized by high professional independence and decision-making in the scope of services provided. Such nurses were licensed to treat wounds and therefore also had the right to independently implement and conduct NPWT. This, in turn, may constitute the basis for revolutionizing the methods for treating wounds in the patient’s home environment through popularization of NPWT at home and as an inpatient [29]. The COVID-19 pandemic was one of the factors that changed the perception of therapeutic and care activities performed in outpatient care settings. It was pointed out that specialist activities should be implemented and expanded, and also that the therapy should be monitored and supervised through teleconsultation, which shortens the time and number of follow-up appointments at the patient’s home. Banasiewicz et al. report that trained nursing staff can, independently or in an interdisciplinary team, conduct therapeutic activities in the patient’s home [30]. Almost a decade earlier, research by Moffatt et al. on the experiences of patients treated for complex, difficult-to-heal wounds with NPWT at home revealed a number of positive psychosocial effects of using this therapy in this environment. The participants of the study perceived NPWT as an active intervention specifically related to the progression of wound treatment while maintaining the ability to perform social roles [31]. A report by Wu et al. [32] also indicates the benefits of transferring patients with NPWT for diabetic foot wounds from medical facilities to the home environment. Based on the research results, the authors clearly emphasize that this method, by means of portable and compact apparatus, may not only bring the expected therapeutic results in terms of healing but also reduce the costs and potential risk of hospitalization of the patient. It should be noted that NPWT conducted at home allows the patient to move freely and function in everyday life while maintaining the continuity of therapy.

With regard to inpatient treatment, surgical departments are in the lead over other medical wards and institutions in terms of activities related to the treatment of wounds of various etiologies. This is largely due to “surgically programmed” wounds resulting from procedures and operations. According to the conducted study, nurses working in surgical wards are characterized by greater knowledge, motivation and readiness to implement NPWT compared to nurses working in nonsurgical medical departments. It can be assumed that this is related to the daily possibility of active participation in the treatment of wounds carried out within the surgical ward. The data obtained make it possible to postulate that the daily work experience and practical activities in the field of NPWT positively influence the perception of this method and is a factor that encourages nurses to implement NPWT independently. 

Nursing in Poland is still in the process of development and nurses have the opportunity to expand their qualifications with more advanced medical services. NPWT used in the treatment of chronic wounds can be implemented and conducted independently and in an interdisciplinary team by nurses in Poland. In our own unpublished material from 2018 to 2022, we independently conducted NPWT in over 100 patients on an outpatient basis with satisfactory results, mainly in the treatment of pressure ulcers, complicated postoperative/traumatic wounds and diabetic foot wounds. Unfortunately, the low popularity and high specificity of this method mean that nurses who have not yet had any practical experience with NPWT declare high readiness to implement this form of therapy; however, they require support and introduction to its use by an experienced clinician.

### Limitations

A small number of doctors (two surgeons) and nurses initially verified the tool. In addition, there is no reference to other study results dealing with the subject matter. This is the first study to present a questionnaire on NPWT perception, but, despite satisfactory results, further research is required.

## 5. Conclusions

The perception of NPWT in the surveyed group of nurses is influenced by numerous factors, including self-assessment of their own knowledge, motivation and readiness to use NPWT. The study group is characterized by a high level of perception of NPWT despite low motivation related to the availability and knowledge of the method. It has not been demonstrated that the professional education obtained would determine the knowledge, motivation and readiness to conduct NPWT. Factors influencing the readiness to undertake NPWT were the place of professional practice and the mentoring support declared. The higher the assessment of the respondents regarding their own knowledge about wound treatment, the higher the assessment of knowledge about wound treatment with NPWT. Practical skills and motivation are indispensable elements that should be met during the training and education of nurses in the field of prevention and treatment of wounds.

## Figures and Tables

**Table 1:** NPWT perception questionnaire construction.

Cronbach Alpha: 0.92					
		Mean	SD	Item–Total Corel	If α Is Omitted
Knowledge	1. NPWT is a method which significantly speeds up the process of chronic wound healing compared to traditional local treatment methods	4.43	0.75	0.52	0.92
	2. NPWT improves the blood supply to the tissues, drains excess exudate, stops the unpleasant smell and shrinks the edges of the wound	4.46	0.77	0.52	0.92
	3. Before NPWT is implemented, necrosis should be removed from the wound (surgically or mechanically) as much as possible	4.41	0.76	0.51	0.92
	4. The optimal negative pressure used in the treatment of chronic wounds with the NPWT method oscillates between the values: 100 to 125 mmHg and is adjusted individually to the patient	3.96	0.90	0.67	0.92
	5. Location of a wound that makes application and maintenance of the dressing difficult is not a criterion for waiving this method	4.05	0.92	0.55	0.92
	6. In case of difficulties in obtaining tightness of the dressing. e.g., due to the location of the wound, additional sealing elements should be used (stoma paste, additional foil)	4.35	0.81	0.53	0.92
	7. Intermediate layer, constituting an active dressing placed under a polyurethane sponge, increases the stimulation of granulation	4.14	0.82	0.53	0.92
	8. If it is impossible to maintain tightness and leakage of the dressing, it should not be kept for more than 2–4 h	3.70	0.97	0.45	0.92
	9. The vacuum dressing can be changed every 3–5 days, if there are no indications for an earlier change	4.17	0.84	0.62	0.92
	10. During NPWT therapy, the patient does not experience any limitations in performing hygienic and rehabilitation activities	3.83	0.98	0.54	0.92
Motivation	11. I am motivated to implement NPWT in practice	4.30	0.79	0.57	0.92
	12. I can count on support (mentor, other specialists) during the implementation of NPWT	3.79	1.09	0.52	0.92
	13. I have no concerns that NPWT could harm a patient with comorbidities. e.g., with diabetes, heart failure	4.06	0.90	0.60	0.92
	14. I am not afraid that I will make a wrong decision to use NPWT and health risk will occur	3.65	1.01	0.65	0.92
	15. I have sufficient knowledge to implement NPWT into practice	3.02	1.26	0.47	0.92
Readiness	16. I have the skills to implement NPWT in practice	2.92	1.26	0.73	0.91
	17. I have the opportunity to consult the patient with other specialists during NPWT	3.14	1.28	0.73	0.91
	18. I can prepare a family and a patient for NPWT therapy	3.06	1.24	0.61	0.92
	19. I can independently dress the wound with a vacuum dressing and set the apparatus appropriately	2.80	1.37	0.71	0.91
	20. I have sufficient knowledge to implement NPWT into practice	3.02	1.26	0.67	0.92

**Table 2:** The key to domain-specific assessment for NPWT readiness in nurses.

**Domain I—Knowledge:** **(α = 0.77)**	**Domain II—Motivation:** **(α = 0.88)**	**Domain III—Readiness:** **(α = 0.94)**	**Total Score** **(α = 0.92)**
10–20 pts.—lack of knowledge	5–10 pts.—lack of motivation	5–10 pts.—lack of readiness	20–40 pts.—lack of readiness
21–30 pts.—low level of knowledge	11–15 pts.—low level of motivation	11–15 pts.—low level of readiness	41–60 pts.—low level of readiness
31–40 pts.—average level of knowledge	16–20 pts.—average level of motivation	16–20 pts.—average level of readiness	61–80 pts.—average level of readiness
41–50 pts.—high level of knowledge	21–25 pts.—high level of motivation	21–25 pts.—high level of readiness	81–100 pts.—high level of readiness

**Domain I—Knowledge:**

**(α = 0.77)**

**Domain II—Motivation:**

**(α = 0.88)**

**Domain III—Readiness:**

**(α = 0.94)**

**Total Score**

**(α = 0.92)**

**Table 3:** Clinical and sociodemographic characteristics of the study participants.

Examined Parameter *n* = 401	
Age (yrs)	44.96 (25–65)
Sex	
Women (*n*)	391 (97.5%)
Men (*n*)	10 (2.5%)
Education (*n*)	
High School (*n*)	59 (14.7%)
BSc (*n*)	97 (24.2%)
MSc (*n*)	293 (73.1%)
Postgraduate (specialization) (*n*)	237 (59.1%)
Specialist courses entitling of wound treatment (*n*)	98 (24.4%)
Place of work	
Own business (*n*)	35 (8.7%)
Primary health care (*n*)	67 (16.7%)
Surgical ward (*n*)	162 (40.4%)
Maintenance ward (*n*)	75 (18.7%)
Long-term care (*n*)	58 (14.5%)
Home hospice (*n*)	46 (11.5%)
Other (*n*)	69 (17.2%)

**Table 4:** Professional experience in terms of the ability to apply a vacuum dressing.

Examined Parameters *n* = 401	
Professional experience of the respondents and independence in terms of the ability to apply a vacuum dressing	
Independent application of a vacuum dressing	48 (12.0%)
Assisting in application of a vacuum dressing	84 (20.9%)
Observing application of a vacuum dressing	54 (13.5%)
Lack of experience	215 (53.6%)

**Table 5:** Results of the NPWT perception questionnaire—total (sum of 3 domains).

Cronbach Alpha: 0.92								
NPWT—Total	Basic Descriptive Statistics							
	N	Mean	Median	Min.	Max.	Quartile I	Quartile III	SD
[20–100 pts]	401	75.77	75.00	37.00	100.00	66.00	86.00	12.72

**Table 6:** The results of the NPWT perception questionnaire (*n* = 401).

	Knowledge (Domain I)		Motivation (Domain II)		Readiness (Domain III)		NPWT	
	N	%	N	%	N	%	N	%
Lack	0	0.0%	3	0.7%	113	28.2%	1	0.2%
Low	27	6.7%	54	13.5%	108	26.9%	52	13.0%
Average	137	34.2%	191	47.6%	106	26.4%	198	49.4%
High	237	59.1%	153	38.2%	74	18.5%	150	37.4%
Total	401	100.0%	401	100.0%	401	100.0%	401	100.0%

**Table 7:** Assessment of the relationship between the level of knowledge and the level of motivation and readiness to implement NPWT.

Variables	Rho	*p*
Knowledge (domain I) and motivation to use NPWT (domain II)	**0.63**	**<0.001**
Knowledge (domain I) and readiness to use NPWT (domain III)	**0.47**	**<0.001**

Rho—value of Spearman’s rank correlation test; *p*—test probability index.

**Table 8:** Assessment of the relationship between the overall readiness to implement NPWT and the age and level of education.

Variables	Rho	*p*
Total NPWT Perception Score and Age	**−0.04**	**0.398**
Total NPWT Perception Score and Age and level of nursing education	**0.03**	**0.507**

Rho—value of Spearman’s rank correlation test; *p*—test probability index.

**Table 9:** Assessment of the relationship between general willingness to implement NPWT and completing a specialist course entitling to wound treatment.

NPWT	With the Course				Without the Course				Z	*p*
	Mean	Me	Min.	Max.	Mean	Me	Min.	Max.		
[20–100 pts]	78.59	81.00	37.00	100.00	74.86	74.00	45.00	100.00	**2.62**	**0.009**

Z—Mann–Whitney U test value; *p*—test probability index.

**Table 10:** Assessment of the relationship between the overall readiness to implement NPWT and work in surgical.

NPWT	Work in Surgical Ward				Does not Work in Surgical Ward				Z	*p*
	Mean	Me	Min.	Max.	Mean	Me	Min.	Max.		
[20–100 pts]	80.80	81.50	56.00	100.00	72.36	71.00	37.00	100.00	**6.56**	**<0.001**

Z—Mann–Whitney U test value; *p*—test probability index.

## Data Availability

The data presented in this study are available on reasonable request from the corresponding author: pwiech@ur.edu.pl.

## References

[1] Kelley A.S., Morrison R.S. (2015). Palliative care for the seriously ill. N. Engl. J. Med..

[2] Atkin L., Bućko Z., Conde Montero E., Cutting K., Moffatt C., Probst A., Romanelli M., Schultz G.S., Tettelbach W. (2019). Implementing TIMERS: The race against hard-to-heal wounds. J. Wound Care.

[3] Sibbald R.G., Williamson D., Orsted H.L., Campbell K., Keast D., Krasner D., Sibbald D. (2000). Preparing the wound bed—Debridement bacterial balance and moisture balance. Ostomy/Wound Manag..

[4] Sibbald R.G., Elliott J.A., Persaud-Jaimangal R., Goodman L., Armstrong D.G., Harley C., Coelho S., Xi N., Evans R., Mayer D.O. (2021). Wound Bed Preparation 2021. Adv. Ski. Wound Care.

[5] Zieliński M., Banasiewicz T., Krasiński Z., Jawień A., Gabriel M. (2017). Efficacy of highly absorbent, polyacrylate fibre and silver-containing lipidocolloid dressings in the inflammatory stage of chronic wound healing process. Leczenia Ran.

[6] Edmonds M., Lázaro-Martínez J.L., Alfayate-García J.M., Martini J., Petit J.M., Rayman G., Lobmann R., Uccioli L., Sauvadet A., Bohbot S. (2018). Sucrose octasulfate dressing versus control dressing in patients with neuroischaemic diabetic foot ulcers (Explorer): An international, multicenter, double-blind, randomized, controlled trial. Lancet Diabetes Endocrinol..

[7] Apleqvist J., Willy C., Fagerdahl A.M., Fraccalveri M., Malmsjo M., Piaggesi A., Probst A. (2017). EWMA Document: Negative Pressure Wound Therapy. J. Wound Care.

[8] Banasiewicz T. (2014). NPWT Sentenced to Success. Negat. Press. Wound Ther..

[9] Dowsett C., Davis L., Henderson V., Searle R. (2012). The economic benefits of negative pressure wound therapy in community-based wound care in the NHS. Int. Wound J..

[10] Meloni M., Izzo V., Vainieri E., Giurato L., Ruotolo V., Uccioli L. (2015). Management of negative pressure wound therapy in the treatment of diabetic foot ulcers. World J. Orthop..

[11] Upton D., Andrews A. (2013). Negative pressure wound therapy: Improving the patient experience. Part 1 of 3. J. Wound Care.

[12] Liu X., Zhang H., Cen S., Huang F. (2018). Negative pressure wound therapy versus conventional wound dressings in treatment of open fractures: A systematic review and meta-analysis. Int. J. Surg..

[13] Révész E.S., Altorjay Á., Montskó V., Hangody L. (2022). Effectiveness of negative pressure wound therapy: Minimum five-year follow-up and review of the literature. Jt. Dis. Relat. Surg..

[14] Bazaliński D. (2019). Skuteczność Terapii Biologicznej z Wykorzystaniem Larw Lucilia Sericata w Leczeniu Ran Przewlekłych u Chorych w Opiece Długoterminowej i Paliatywnej. [Effectiveness of Biological Therapy Using Lucilia Sericata Larvae in the Treatment of Chronic Wounds in Patients in Long-Term and Palliative Care].

[15] Huang Y., Hu J., Mao B., Ni P., Shou Y., Hou L., Xie T. (2022). Perspectives on the Process of Negative Pressure Wound Therapy at Home in Patients with Chronic Wound: A Qualitative Descriptive Study. Int. J. Low. Extrem. Wounds.

[16] Szewczyk M.T., Cwajda-Białasik J., Mościcka P., Cierzniakowska K., Bazaliński D., Jawień A., Spannbauer A., Polak A., Sopata M., Kozłowska E. (2020). Treatment of pressure ulcers—Recommendations of the Polish Wound Management Association. Part II. Leczenie Ran.

[17] Bazaliński D., Zmora M., Przybek-Mita J., Kózka M. (2017). The impact of nurses’ qualifications on their knowledge in the field of prevention and treatment of pressure ulcers. Pielęgniarstwo Chir. Angiol./Surg. Vasc. Nurs..

[18] Norman G., Goh E.L., Dumville J.C., Shi C., Liu Z., Chiverton L., Stankiewicz M., Reid A. (2020). Negative pressure wound therapy for surgical wound healing by primary closure. Cochrane Database Syst. Rev..

[19] Xie W., Dai L., Qi Y., Jiang X. (2022). Negative pressure wound therapy compared with conventional wound dressings for closed incisions in orthopedic trauma surgery: A meta-analysis. Int. Wound J..

[20] Huang Y., Mao B., Hu J., Xu B., Ni P., Hou L., Xie T. (2021). Consensus on the health education of home-based negative pressure wound therapy for patients with chronic wounds: A modified Delphi study. Burns Trauma.

[21] Głowacz J., Szwamel K. (2022). Nursing staff’s knowledge of chronic wounds and methods of their treatment. Pielęgniarstwo Chir. Angiol./Surg. Vasc. Nurs..

[22] Monsen C., Acosta S., Kumlien C. (2017). Patients experiences of negative pressure wound therapy at home for the treatment of deepperivascular groin infection after vascular surgery. J. Clin. Nurs..

[23] Bolas N., Holloway S. (2012). Negative pressure wound therapy: A study on patient perspectives. Br. J. Community Nurs..

[24] Bazaliński D., Przybek Mita J., Ścisło L., Więch P. (2022). Perception and Readiness to Undertake Maggot Debridement Therapy with the Use of Lucilia sericata Larvae in the Group of Nurses. Int. J. Environ. Res. Public Health.

[25] Bazaliński D., Kózka M., Karnas M., Więch P. (2019). Effectiveness of Chronic Wound Debridement with the Use of Larvae of Lucilia Sericata. J. Clin. Med..

[26] Morozov A.M., Sherman R.A. (2019). Survey of patients of the Tver region of Russia regarding maggots and maggot therapy. Int. Wound J..

[27] Steenvoorde P., Buddingh T.J., van Engeland A., Oskam J. (2005). Maggot therapy and the “yuk” factor: An issue for the patient?. Wound Repair Regen..

[28] Skórka M., Malisiewicz A., Sałacińska I., Bazaliński D. (2021). The mentoring scheme in clinical nursing involving chronic wound treatment, as an efficient tool for professional development—A study of 3 cases. Nurs. Probl..

[29] Michalik A., Kolonko J., Maciejewski D., Kadłubowska M. (2020). Patient with a consideration or confirmation of COVID-19—What every nurse should know. Pielęgniarstwo Chir. Angiol./Surg. Vasc. Nurs..

[30] Banasiewicz T., Becker R., Bobkiewicz A., Fraccalvieri M., Francuzik W., Hutan M., Laukoetter M., Malka M., Mańkowski B., Szentkereszty Z. (2020). Prevention and therapy of acute and chronic wounds using NPWT devices during the COVID-19 pandemic, recommendation from the NPWT Working Group. Negat. Press. Wound Ther. J..

[31] Moffatt C.J., Mapplebeck L., Murray S., Morgan P.A. (2011). The experience of patients with complex wounds and the use of NPWT in a home-care setting. J. Wound Care.

[32] Wu S.C., Armstrong D.G. (2008). Clinical outcome of diabetic foot ulcers treated with negative pressure wound therapy and the transition from acute care to home care. Int. Wound J..

